# A Nuclease from *Streptococcus mutans* Facilitates Biofilm Dispersal and Escape from Killing by Neutrophil Extracellular Traps

**DOI:** 10.3389/fcimb.2017.00097

**Published:** 2017-03-28

**Authors:** Jia Liu, Luping Sun, Wei Liu, Lihong Guo, Zhaohui Liu, Xi Wei, Junqi Ling

**Affiliations:** Guangdong Provincial Key Laboratory of Stomatology, Guanghua School of Stomatology, Hospital of Stomatology, Sun Yat-Sen UniversityGuangzhou, China

**Keywords:** *Streptococcus mutans*, neutrophil extracellular traps, nuclease, biofilm dispersal, immune defense

## Abstract

*Streptococcus mutans* is the primary etiologic agent of dental caries and occasionally infective endocarditis, with the ability to form biofilms and disperse cells into distal sites to exacerbate and spread infection. In this study, we identified a nuclease (DeoC) as a *S. mutans* biofilm dispersal modulating factor through microarray analysis. *In vitro* assays revealed a dispersal defect of a *deoC* deletion mutant, and functional studies with purified protein were indicative of the biofilm dispersal activity of DeoC. Neutrophils are a key host response factor restraining bacterial spreading through the formation of neutrophil extracellular traps (NETs), which consist of a nuclear DNA backbone associated with antimicrobial peptides. Therefore, we hypothesized that the dispersed *S. mutans* might utilize DeoC to degrade NETs and escape killing by the immune system. It was found that *S. mutans* induced NET formation upon contact with neutrophils, while the presence of NETs in turn enhanced the *deoC* expression of *S. mutans*. Fluorescence microscopy inspection showed that *deoC* deletion resulted in a decreased NET degradation ability of *S. mutans* and enhanced susceptibility to neutrophil killing. Data obtained from this study assigned two important roles for DeoC in *S. mutans*: contributing to the spread of infection through mediating biofilm dispersal, and facilitating the escape of *S. mutans* from neutrophil killing through NET degradation.

## Introduction

*Streptococcus mutans*, the primary etiologic agent of human dental caries (Ahn et al., 2006), and occasionally infective endocarditis (Jung et al., 2009), can form biofilms on the surface of teeth, referred to as dental plaque biofilm. Unlike other pathogens that produce virulence factors that can wreak havoc in the host, *S. mutans* has evolved to exist as part of the normal elements of the oral dental biofilm (Smith and Spatafora, 2012). Thus, *S. mutans* can easily gain access into the bloodstream during dental surgery, and colonize injured heart valves and induce endocarditis (Moreillon and Que, 2004) As the major component of biofilm, the extracellular matrix holds bacterial cells together, and decreases their susceptibility to killing by immune defenses and antimicrobial agents (Ahn et al., 2008). The protective lifestyle of biofilm makes *S. mutans*-associated infections difficult or impossible to eradicate and leads to the persistence of chronic infections. Beyond contributing to recalcitrance to eradication mechanisms, biofilms also play an important role in the exacerbation and spread of infection within a host through dispersing bacterial cells into the environment to colonize distal sites and cause new infections (Kaplan, 2010). Proposed biofilm active dispersal strategies utilized by phylogenetically diverse bacteria include production of various extracellular enzymes to degrade the extracellular matrix, like glycosidases, proteases, and nucleases (Lister and Horswill, 2014). The nuclease has been proved to be an endogenous mediator of biofilm dispersal of *Staphylococcus aureus* and a thermonuclease-deficient mutant strain of *S. aureus* exhibited increased biofilm formation (Mann et al., 2009). A potent nuclease of nontypeable *Haemophilus influenzae* has also been implicated in biofilm remodeling and organism dispersal (Cho et al., 2015).

When bacterial cells disperse from the protective biofilm and re-enter the planktonic state, they encounter killing by the host immune defense system (Wartha et al., 2007). Neutrophils are known to be a key host response factor to bacterial challenge. Upon microbial infection, neutrophils are recruited to the infectious site and release neutrophil extracellular traps (NETs) to restrain bacterial spreading (Brinkmann et al., 2004). NETs consist of nuclear DNA as a backbone studded with antimicrobial compounds like histone, calprotectin, and serine proteases, and therefore entrap and kill various microbes (Urban et al., 2006). The nuclear backbone is bactericidal through chelating cations and disrupting bacterial membrane integrity. In response to the entrapment by NETs, microbes have evolved various ways to escape from such neutrophil-associated killing (Halverson et al., 2015). The major strategies used by pathogenic bacteria to evade neutrophil defenses include avoiding contact, preventing phagocytosis, inducing cell death, and evading killing by NETs (Urban et al., 2006). Nucleases have been found in several pathogenic bacteria that facilitate escape from NETs through degrading the DNA backbone of the traps; thus promoting resistance against killing by neutrophils (Thammavongsa et al., 2013). In *Staphylococcus* and *Streptococcus* strains, nucleases have previously been implicated in degrading NETs *in vitro and in vivo. Streptococcus pyogenes* expresses the extracellular DNase Sda1, which increases resistance against killing by human neutrophils and in whole mouse blood (Buchanan et al., 2006). Therefore, two possible roles can be proposed for nucleases during infection, the regulation of biofilm dispersal and the escape of dispersed cells from killing by the immune system. In *S. mutans* GS5, the *deoC*-encoded product could catabolize both homospecific and heterospecific extracellular DNA (Han et al., 2004). In the present study, we initially identified in the *S. mutans*UA159 genome sequence a *deoC* homolog that was closely linked to biofilm dispersal. The presence of a putative nuclease in *S. mutans* intrigued us and led us to investigate whether the nuclease facilitated subsequent escape of dispersed cells from neutrophil entrapment.

## Materials and methods

### Bacterial strains and culture conditions

The bacterial strains, plasmids, and primers used are listed in Table S1. *S. mutans* UA159 was grown at 37°C under anaerobic conditions (90% N_2_, 5% CO_2_, 5% H_2_) in BHI broth (Difco, Sparks, MD, USA). For biofilm formation, 1% sucrose was included in the BHI medium. *Escherichia coli* strains were grown in Luria–Bertani (LB) medium (Difco, Sparks, MD, USA). If required, antibiotics and other supplements were used at the following final concentrations: spectinomycin (Spe), 800 μg/ml; kanamycin, 50–100 μg/ml. The competence stimulating peptide (CSP) was synthesized by Life Invitrogen (Shanghai, China).

### Biofilm dispersal determination

Biofilms were grown in a flow cell (model FC91; BioSurface Technologies, Corp., Bozeman, MT, USA) with connective tubing draining the effluent into a container as described previously (Fux et al., 2004). Overnight culture of *S. mutans* was resuspended in BHI–1% sucrose and a 2 ml inoculum [5 × 10^5^colony forming units (CFUs)/ml] was injected into the inoculation port containing polystyrene (PLS) blocks (VWR Scientific, CA, USA). Bacteria were allowed to attach on PLS blocks for 45 min before a continuous flow of 1 ml/min at 37°C was started. The flowthrough was collected, and the dispersed cells were harvested by centrifugation and resuspended in PBS. The amount of dispersed cells was determined by plate counting and presented as the log (CFU/ml). The remaining adherent biofilm on the PLS blocks was stained with Live/Dead BacLight (Molecular Probes, Invitrogen, Carlsbad, CA, USA) and scanned by confocal laser scanning microscope (LSM 710; Zeiss, Jena, Germany), followed with COMSTAT program analysis (Liu et al., 2012).

### Microarray and bioinformatics analysis

Biofilms at different dispersal stages were collected, suspended in lysis buffer (30 mM Tris-HCl, 1 mM EDTA, 20 mg/ml lysozyme, pH 8.0) and incubated at 37°C with gentle agitation for 40 min. Total RNA was extracted using an RNeasy Mini kit (Qiagen, Dusseldorf, Germany). The RNA obtained from each sample was quantified using the NanoDrop ND-2000 and the RNA integrity was assessed using standard denaturing agarose gel electrophoresis. For microarray analysis, the *S. mutans* Whole Genome Oligo Array (8 × 15K; Agilent Technologies) and the Agilent Array platform were employed. The sample preparation and microarray hybridization were performed based on the manufacturer's standard protocols (http://www.agilent.com). Raw data were processed and normalized using GENESPRING GX software. Microarray data have been deposited at NCBI-GEO (accession no. GSE95377). Genes with a fold change >1.5 and a *P* < 0.05 were selected for further gene expression pattern discovery. The protein–protein interactions for the differentially expressed genes (DEGs) were analyzed through Cytoscape software (Institute of Systems Biology, Seattle, WA, USA). Functional annotation of the DEGs was performed using gene ontology (GO) analysis.

### Construction of a *deoC* deletion mutant and complementation

The whole *deoC* gene was replaced with a Spe-resistance cassette. Approximately 800 bp upstream and downstream, the arms of *deoC* were PCR amplified. The upstream homology arm contained *Xho*I at the 5′ end and *Hin*dIII at the 3′ end, and the downstream homology arm contained *Nde*I at the 5′ end and *Spe*I at the 3′ end. The homology arms were ligated into pFW5 (Podbielski et al., 1996), flanking the Spe-resistance cassette in the multiple cloning site. The resulting plasmid was used to transform *S. mutans* with exogenous CSP added, and transformants were screened on BHI plates containing 800 μg/ml Spe. The sequences of mutant transformants were confirmed by PCR and DNA sequencing. The complementation construct containing the *deoC* ORF with the putative promoter was cloned into the MCSI sites of pFW5. The construct was used to transform the *S. mutans deoC* mutant. Transformants without the antibiotic-resistance phenotype were screened for complementation. The confirmed complemented strain was named pFW5-*deoC*com.

### DNA degradation activity assay and biofilm dispersal study

The DNA degradation activity of *S. mutans* biofilm supernatant was determined using radical enzyme diffusion assays (Macanovic and Lachmann, 1997). In brief, the culture supernatants of *S. mutans* biofilms (48–60 h, 60–72 h, 72–84 h, and 84–96 h) were collected by centrifugation at 8,000 *g* for 15 min, and concentrated in polyethyleneglycol 20,000 (Merck). A 10 μl aliquot of the supernatant was added into the well (diameter = 3 mm) of an agarose plate containing 2 mg/ml salmon sperm DNA, 0.1% Gold View I, 1 mM MgCl_2_, and 1 mM CaCl_2._After overnight incubation, 0.05 M EDTA was added to the plate to stop the reaction. The plate was photographed with a UV gel imaging system (FluorChem Q; ALPHA, USA) and the diameters of the circles of hydrolyzed DNA were analyzed with Image-Pro Plus 6.0.

Biofilm dispersal of *S. mutans* strains was examined as described elsewhere (Kaplan and Fine, 2002). The method employed the microcurrents caused by edge evaporation in an open 100 mm culture dish. *S. mutans* strains (10^3^ CFU/ml) were incubated overnight at 37°C under 85% humidity and 5% CO_2_ for 24 h in the dish. The medium was discarded, and the plate was stained with crystal violet, followed with washing with distilled water and observation by microscopy.

### Cloning, expression, and purification of recombinant proteins

The *S. mutans deoC* gene was amplified from genomic DNA using PCR (TaKaRa) with appropriate primers (Table S1) and cloned into the pET28a expression vector to produce recombinant vector pET-*deoC*. The vector was used to transform *E. coli* BL21 (DE3) cells. The *E. coli* cells were grown in LB medium containing 100 μg/ml kanamycin at 37°C for 1 h, followed by plating on an agar plate with 50 μg/ml kanamycin. A single colony was inoculated into LB medium and cultured at 16°C after the addition of 0.3 mM (final concentration) isopropyl-β-D-thiogalactopyranoside to induce protein expression. The overnight culture was collected for recombinant DeoC protein purification as described previously (Gerner et al., 2016). In brief, the cell pellets were resuspended in lysis buffer (20 mM Tris, 500 mMNaCl, pH 8.0), lysed using an Ultrasonic Cell Disruptor (VCX130, 3 mm; Sonics, USA) for 120 × 4 s and cellular debris was removed by centrifugation at 12,000 *g* for 25 min at 4°C. The recombinant protein was captured on a Ni-IMAC column with loading buffer I (20 mM Tris, 500 mMNaCl, pH 8.0), followed by extensive washing with more than 20 column volumes of loading buffer with 200 mM imidazole. Eluted fractions were collected and separated by SDS-PAGE. Purified proteins were examined by SDS-PAGE and protein concentration was determined using RC and DC Protein Assay (Bio-Rad, CA, USA) by measuring spectrophotometric absorbance at 280 nm.

### Characterization of recombinant protein

Enzyme activity was determined by cleavage of 2-deoxyribose-5-phosphate (DR5P). The reaction mixture contained 1 mM DR5P, 0.5 mM NADH, and 10 μl diluted recombinant protein in a 200 μl total volume (You et al., 2012). The catalyzing assay was conducted at different pH (3.0–10.0) and temperature (10°–70°C) values to analyze the effects of pH and temperature on purified protein activities. The decrease in the NADH level was monitored at 340 nm.

The biofilm dispersal ability of recombinant protein was determined as described previously (Minh Tran et al., 2016). *S. mutans* biofilm was preformed in a 24-well plate and spent medium was aspirated, followed with washing once with PBS. Different concentrations of purified protein were added to the biofilm and plates were incubated at 37°C for 1 h. The remaining biofilm was stained with 0.01% crystal violet, washed twice with PBS buffer, and imaged with a Leica light microscope (Leica Biosystems, Buffalo Grove, IL, USA). To quantify the amount of remaining biofilm, 95% ethanol was added into the wells and the absorbance at 590 nm was determined after 20 min incubation (Minh Tran et al., 2016). Different concentrations of DNase I (Sigma, St Louis, MO, USA) were used as a positive control.

DNA degradation activity of *S. mutans* strain supernatants and the purified protein was determined as described previously (Berends et al., 2010). Briefly, the overnight culture supernatant of *S. mutans* strains was collected by centrifugation at 8,000 *g* for 15 min, and concentrated in polyethyleneglycol 20,000 (Merck). A 5 μl aliquot of the supernatant or the purified protein was incubated with salmon sperm DNA for 1 h at 37°C. The nuclease reaction was stopped with 12.5 μl 0.33 M EDTA (pH 8.0) and the sample was run in a 1% agarose gel for visual examination of DNA degradation.

### Isolation of human neutrophils

Human venous blood was obtained from healthy volunteers according to the recommendations of the local ethical committee (ERC-2015-14) and written informed consent was provided by all the participants. Neutrophils were purified by centrifugation (1,800 *g*) at room temperature for 30 min with BD Vacutainer CPT™ tubes with sodium citrate, according to the manufacturer's instructions. After centrifugation, the polyester gel was removed and cells in the granulocyte layer were collected, followed by erythrocyte lysis, washing with PBS twice, and resuspension in RPMI 1640 with 10% FBS. The purity of the obtained cells was verified by staining and observation with a microscope.

### NET visualization

The neutrophil cell suspension in RPMI 1640 with 10% FBS was seeded on coverslides (13 mm) coated with poly-L-lysine (Sigma) in a 24-well plate (3 × 10^5^ cells per well) and incubated for 30 min to allow the cells to attach to the coverslides. To chemically induce NET formation, cells were treated with 100 nM phorbol 12-myristate 13-acetate (PMA; Sigma) for 4 h. Untreated or PMA-treated neutrophils were exposed to *S. mutans* UA159 wild type, *deoC* mutant, or pFW5-*deoC*com infection at a multiplicity of infection of 200 (MOI = 200) for 4 h. The effect of commercial DNaseI on the NET formation was also investigated as a control. Cells on the coverslides were fixed with 4% paraformaldehyde for 10 min, followed by treatment with 1% Triton X-100 for 10 min and 5% bovine serum albumin (Sigma) block for 1 h. The cells were then incubated with the primary antibody anti-neutrophil elastase (anti-NE; abcam68672) and a secondary antibody conjugated to HRP (abcam6721) to visualize the NE, and the DNA backbone of NETs was detected with DAPI. Specimens were mounted in anti-fade fluorescence medium (Life Invitrogen) and observed with an oil immersion objective fluorescent microscope (Olympus BX63).

### Measurement of reactive oxygen species (ROS)

ROS production of human neutrophils was measured by a luminometric assay as described previously (Seper et al., 2013). Briefly, neutrophils (3 × 10^4^cells per well) were seeded in a black 96-well plate, followed with addition of 10 μM2, 7-dichlorodihydrofluorescein diacetate. PMA (100 nM) and the *S. mutans* strain (MOI = 200) were added to stimulate the formation of NETs, and untreated neutrophils were used as a control. Chemiluminescence was determined every 10 min for 2 h in a POLARstar Omega microplate reader (BMG Labtech, Ortenberg, Germany). The ROS production was shown as the area under the curve or as relative light units (RLU).

### Extracellular DNA fluorescence assay

NET formation was measured by fluorescence assay as described previously (Juneau et al., 2015). Briefly, neutrophils (3 × 10^4^cells per well) were seeded in a black 96-well plate and cell-impermeable Sytox Green DNA-binding dye (5 μM) was added. Cells were stimulated either with 100 nM PMA or *S. mutans* strains (MOI = 200), or left untreated, and cells were lysed with 1% Triton X-100 as a 100% control. Fluorescence was monitored every 30 min for a period of 9 h in a POLARstar Omega microplate reader (BMG Labtech, Ortenberg, Germany). As a control for DNA degradation, DNase I (100 U/ml) was added after 5 h to PMA- or *S. mutans* strain-stimulated neutrophils. NET formation was normalized to total neutrophil DNA obtained by Triton X-100 lysis and presented as percentage of DNA fluorescence compared with the 100% lysis control.

### Bacterial survival assay and determination of *deoC* expression

Neutrophils (3 × 10^4^ per well) were seeded in a 24-well plate and stimulated with 100 nM PMA (37°C, 5% CO_2_) for 4 h to induce NET formation. Cells were divided into three groups, treated with 10 μg/ml cytochalasin D (Sigma) to inhibit phagocytic killing, cytochalasin D, and DNase I (100 U/ml), and the same amount of RPMI 1640 medium as a control, for 15 min. The *S. mutans* strains were added with an MOI of 200, followed by centrifugation at 800 *g* for 10 min to get the bacteria in contact with the NETs formed (Berends et al., 2010; Seper et al., 2013). After incubation for 4 h, DNase I was added to the wells not having received DNase I for 20 min to release the NET-entrapped bacteria. Well contents were serially diluted and plated for CFU enumeration. Bacterial survival was expressed as a percentage of the initial inoculum per well (Juneau et al., 2015). To visualize *S. mutans* entrapment, the co-cultured neutrophils and *S. mutans* strains on PLS blocks were fixed with 2.5% glutaraldehyde, followed with dehydration in ascending concentrations of ethanol and drying to the critical point. The samples were sputter coated with gold-palladium and observed by scanning electron microscopy (Tran et al., 2016).

To measure gene expression of *deoC* in *S. mutans* when entrapped in NETs, *S. mutans* was collected for RNA extraction and cDNA synthesis was performed with a SuperScript cDNA Synthesis kit (Invitrogen) using 200 ng total RNA. Quantitative real-timePCR (qRT-PCR) was used for quantification of *deoC* expression with 16S rRNA as the internal control. The reaction program was 95°C for 2 min, followed by 40 cycles of 95°C 15 s and 60°C for 30 s. Threshold cycle (CT) values were determined and data were analyzed with the 2^−ΔΔCT^ method. *S. mutans* wild-type strain incubated with salmon sperm DNA (Sigma) was used as the positive control and *S. mutans* incubated without neutrophils or DNA served as the internal control.

### Statistical analysis

Data analyses were performed using SPSS 13.0, and differences among groups were analyzed by ANOVA, followed by Tukey's multiple comparison tests. Results were shown as mean values ± standard deviation and considered statistically significant at *P* < 0.05.

## Results

### Dispersal dynamics of *S. mutans* biofilm

**Figure 1:**
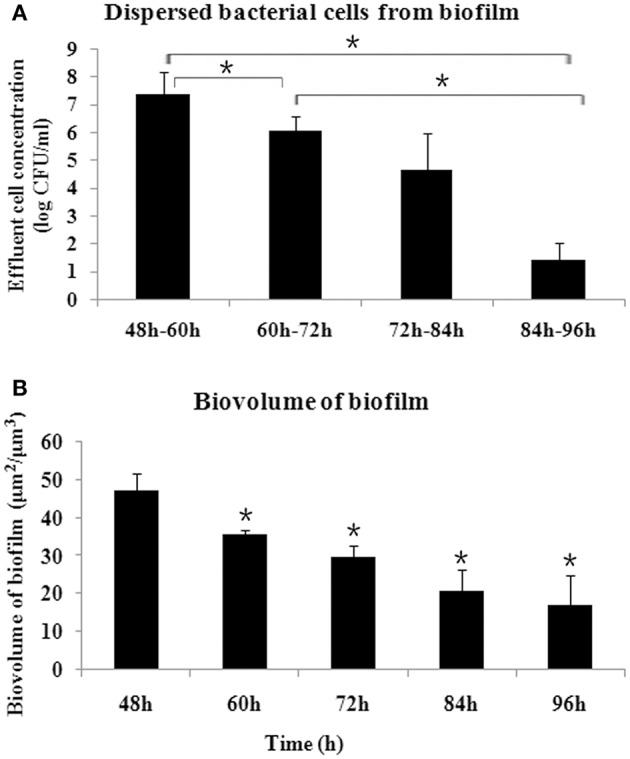
**Dynamics of ***S. mutans*** biofilm dispersal. (A)** Amount of dispersed cells every 12 h, as determined by quantifying the number of cells in the flowthrough, presented as log (CFU/ml). More cells were dispersed during 48–60 h compared with the other time periods, and the amount of dispersed cells decreased after 84 h (^*^P < 0.05). **(B)** Dynamics of biovolume of remaining *S. mutans* biofilm after dispersal, determined by confocal laser scanning microscope and analyzed by COMSTAT. ^*^Significant difference compared with 48 h (*P* < 0.05).

The biofilm released bacterial cells during the examined time periods and the amount of bacterial cells was determined every 12 h. As shown in Figure 1A, during the time periods of 48–60 h, more cells were dispersed from biofilm compared with the other time periods (*P* < 0.05), representing a crucial stage for biofilm dispersal. The biofilm dispersal became steady, with no significant difference in the amount of dispersed cells observed, between 60–72 h and 72–84 h (*P* > 0.05). During 84–96 h, the amount of dispersed cells decreased significantly (*P* < 0.05). Corresponding to the dispersal, the biomass of biofilm decreased gradually and reached a steady state after 84 h (Figure 1B).

### Global transcriptional changes during *S. mutans* biofilm dispersal

We used whole-genome microarrays to identify the global transcriptional changes during biofilm dispersal and also to investigate the molecular mechanism regulating biofilm dispersal. Based on the data shown in Figure 1, 48–60 h represented a rapid biofilm dispersal stage and at 96 h the dispersal greatly decreased. Therefore, the transcriptome of 96 vs. 60 h biofilm and 60 vs. 48 h biofilm was compared. A total of 160 genes were identified as differentially expressed, with 26 genes up-regulated (Table S2) and 134 genes down-regulated in the 96 vs. 60 h biofilm group (Table S3). In the 60 vs. 48 h group, 137 genes (30 up-regulated and 107 down-regulated) were differentially expressed, as shown in Tables S4, S5.

**Figure 2:**
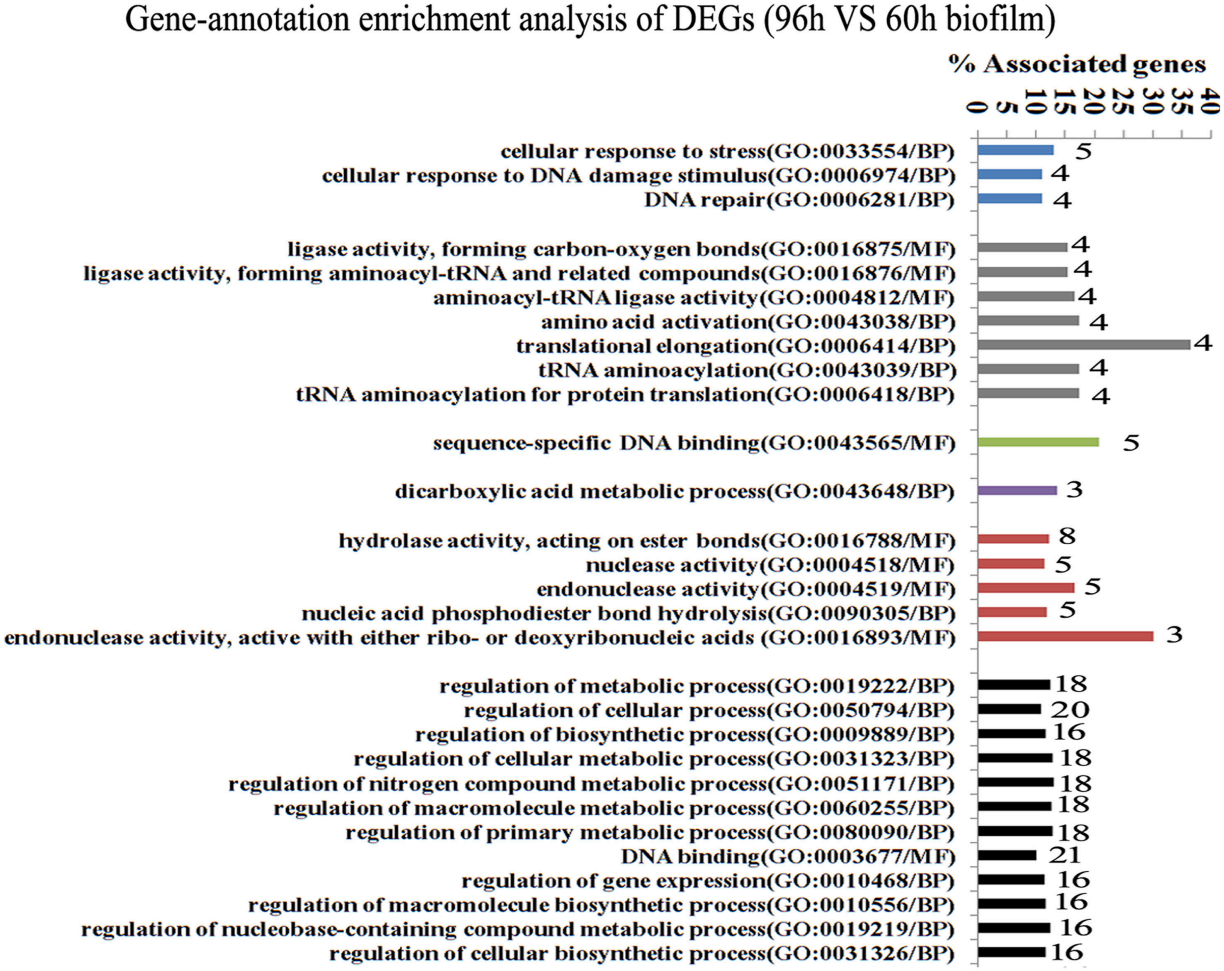
**Transcriptome analysis of ***S. mutans*** biofilm during dispersion (96 vs. 60 h)**. Gene ontology enrichment analysis of the DEGs (96 vs. 60 h) was obtained based on protein network analysis. Six interaction networks represented by different colors were identified among 134 DEGs. The DEGs in GO term translational elongation and in GO term endonuclease activity reached 36.6 and 30%; GO, gene ontology; BP, biological process; MF, molecular function.

**Figure 3:**
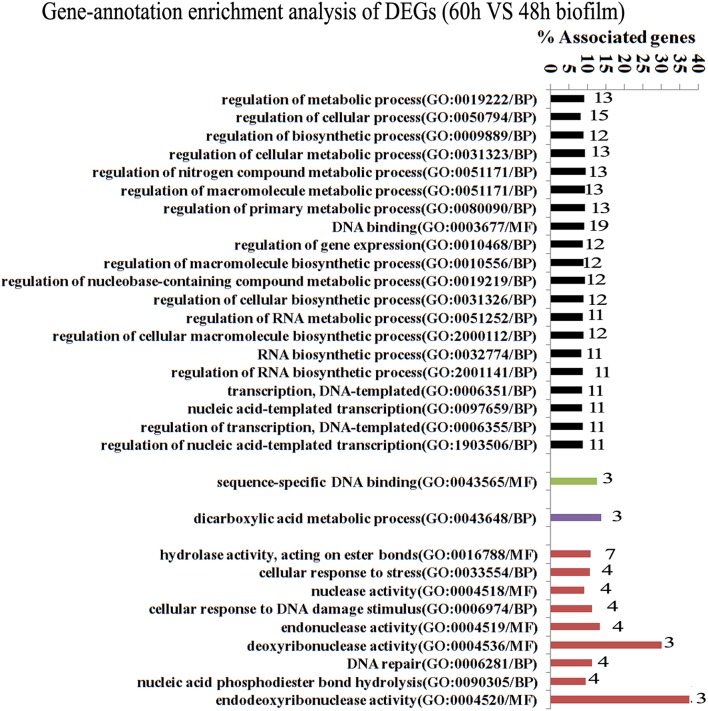
**Transcriptome analysis of ***S. mutans*** biofilm during dispersion (60 vs. 48 h)**. Four protein interaction networks represented by different colors were identified among 137 DEGs. The genes in each network were clustered into functional annotation terms. The DEGs in GO term deoxyribonuclease activity and endodeoxyribonuclease activity reached 30 and 37.5%. GO, gene ontology; BP, biological process; MF, molecular function.

Since gene interaction networks are important for various biological processes, we analyzed protein interactions among the identified DEGs. In the 96 vs. 60 h group, the DEGs were clustered in 6 interaction networks (Figure 2). Four of the interaction networks were also predicted in the 60 vs. 48 h biofilm group (Figure 3). It seemed that the DEGs formed a highly linked network, which indicated that the genes in the identified interaction networks might be associated with biofilm dispersal.

GO enrichment analysis was then carried out to identify pathways that may regulate *S. mutans* biofilm dispersal based on the identified interaction networks. In total, 37 GO terms were assigned in the 96 vs. 60 h group, among which the DEGs with GO term translational elongation and with GO term endonuclease activity reached 36.6 and 30%, respectively (Figure 2). Among the 31 GO terms in the 60 vs. 48 h group, the DEGs in GO term deoxyribonuclease activity and endodeoxyribonuclease activity reached 30 and 37.5%, respectively (Figure 3). The overlap of DEGs in GO terms deoxyribonuclease activity and endodeoxyribonuclease activity indicated that nuclease activity might be critical to biofilm dispersal.

To validate the microarray data, we randomly selected 20 genes for expression analysis by qRT-PCR using 16S rRNA as the internal control. As shown in Figure S2, most of the selected genes showed a similar expression trend, which supported a strong level of confidence in our microarray data.

### *deoC* mutant exhibits compromised dispersal ability

**Figure 4:**
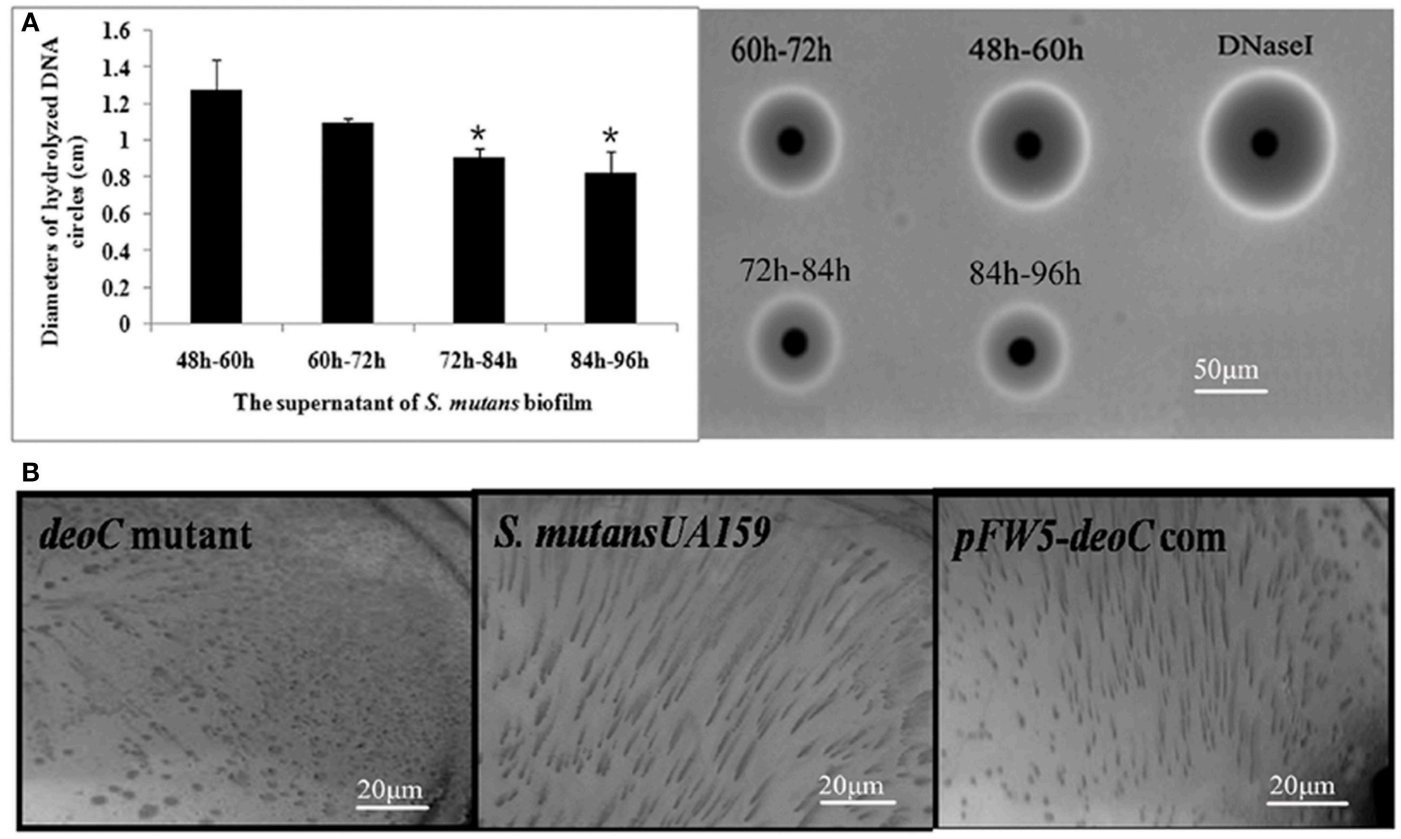
**The ***deoC*** mutant exhibited compromised dispersal ability. (A)** Nuclease activity of *S. mutans* biofilm supernatant. The nuclease activity reached the maximum at 48–60 h, characterized by the largest diameter of DNA hydrolysis circle. Data are presented as mean ± SD,^*^P < 0.05 compared with 48–60 h. **(B)** *deoC* was responsible for *S. mutans* biofilm dispersal. Comet tails caused by the release of cells from microcolonies/nascent biofilms were observed inthe *S. mutans* wildtype and *deoC* complemented strain, but no evidence of dispersal was noted in the *deoC* mutant.

To study whether *deoC* played a potential role in biofilm dispersal, we examined the DNase activity of *S. mutans* biofilm supernatants at different stages. As shown in Figure 4A, the supernatant of the 48–60 h period exhibited the highest level of DNase activity characterized by the larger diameter compared with the 72–84 h and 84–96 h periods (*P* < 0.05), which was in accordance with the dispersal extent of biofilm in Figure 1. We thus speculated that this protein may initiate the biofilm dispersal. To verify this speculation, we constructed a *deoC* deletion mutant and compared the dispersal ability of the mutant with the wild-type strain through the biofilm dispersal experiment. The *deoC* deletion mutant was verified by PCR and sequencing (Figure S1). In the dispersal assay, microturbulence in the plates causes a comet-like tail to form as organisms disperse from the nascent microcolony. The dispersal was noted by the comet tail of satellite colonies formed by *S. mutans* wild type (Figure 4B). However, only a few of the microcolonies exhibited a similar comet tail in the plates containing the *deoC* mutant. These data strongly suggested that DeoC was involved in the biofilm dispersal of *S. mutans*.

### *deoC* activity in *S. mutans*

**Figure 5:**
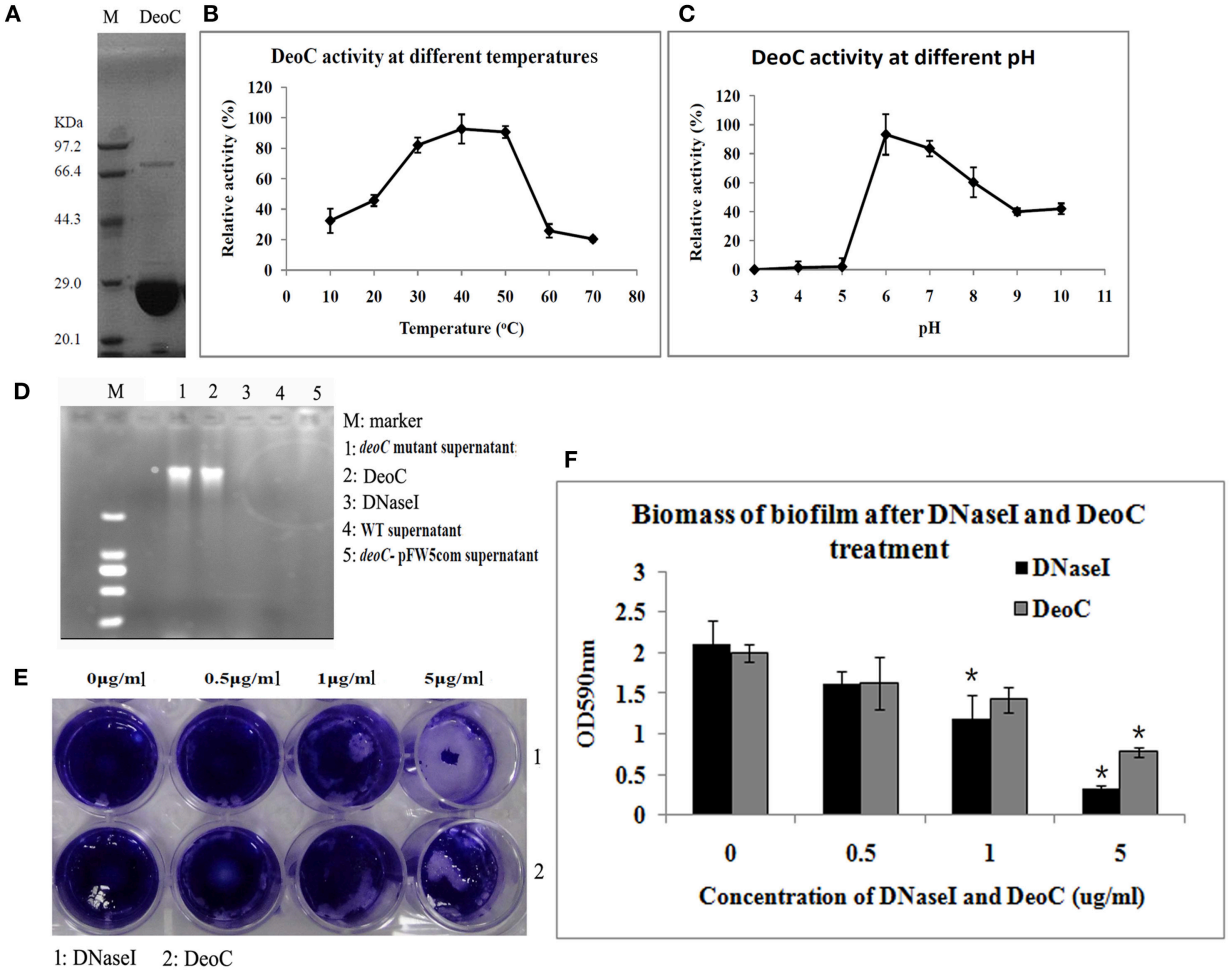
**Characteristics of DeoC in ***S. mutans***. (A)** His-tagged DeoC was expressed and purified from *E. coli* and was analyzed by SDS-PAGE. Lane M, protein molecular mass marker. **(B,C)** Activity of purified DeoC at different pHs and temperatures. **(D)** Degradation of DNA by the supernatant of *S. mutans* strains. **(E,F)** Exogenous application of DeoC disrupted *S. mutans* biofilm. Preformed biofilm was incubated at 37°C for 1 h with the indicated concentrations of DeoC and stained with crystal violet. ^*^Significant decrease in biofilm biomass after 5 μg/ml DeoC treatment (*P* < 0.05).

To confirm DeoC activity, we expressed and purified the DeoC His-tagged recombinant protein (Figure 5A). In order to determine the optimal conditions for the catalytic activity, we quantitatively assayed the effects of pH and temperature on purified DeoC activities. The results showed that purified DeoC was capable of catabolizing DR5P and was optimally active at pH 6.0 (Figure 5B) and 50°C (Figure 5C), but could not degrade DNA independently (Figure 5D). The DNA degradation activity of culture supernatants of *S. mutans* was also investigated via degradation of salmon sperm DNA. The *deoC* mutant supernatant demonstrated no DNA degradation activity, indicating that DeoC played an indispensable role in nuclease activity of *S. mutans*. In contrast, supernatants from the wild-type strain and DNaseI efficiently degraded DNA marker (Figure 5D). Degradation of DNA could be restored in the complemented strain; thus, excluding polar effects of mutagenesis.

To determine whether DeoC could disperse preformed biofilm, we treated biofilm with increasing concentrations of purified DeoC, and commercial DNaseI was used as a positive control. The biofilm was progressively disrupted as the DeoC concentration increased, and a significant decrease in biofilm biomass was observed when the DeoC concentration increased to 1μg/ml (*P* < 0.05) compared with the untreated control (Figures 5E,F).

### *S. mutans* induces NET formation and degrades NETs through *deoC*

**Figure 6:**
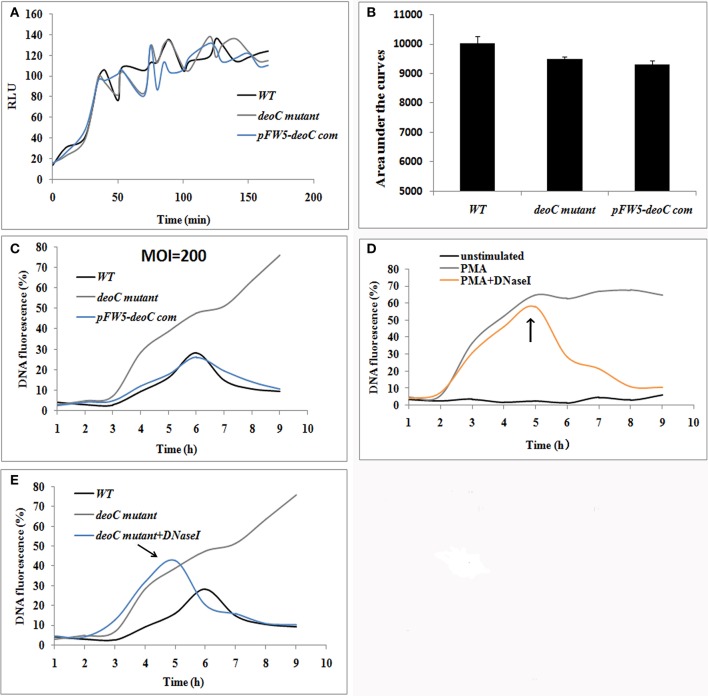
*****S. mutans***-stimulated NET formation of neutrophils. (A,B)** ROS production of human neutrophils by a luminometric assay. ROS production reached a maximum at approximately 2 h (MOI = 200) in all the tested strains, indicative that *S. mutans* was recognized by human neutrophils and had the potential to induce NET formation **(A)**. No significant difference was observed in the ROS levels between the wild type (WT) and *deoC* mutant represented by the areas under the curve **(B)**, indicating that the neutrophil activation potential of *S. mutans* was independent of DeoC. RLU, Relative light units. **(C)** DNA release of neutrophils co-cultured with *S. mutans* strains. **(D)** DNA release of neutrophils stimulated with PMA. DNA release was quantified by the live cell impermeant fluorescent DNA dye SytoxGreen. Data are shown as percentages of DNA fluorescence compared with a TritonX-100 lysis control (100%). **(E)** DNaseI degraded the released DNA of neutrophils induced by *deoC* mutant. DNaseI was added after 5 h of co-culture. The arrows indicated the addition of DNaseI at 5 h.

The oxidative burst is a key feature for neutrophils, which is involved in signaling processes and microbial killing. Thus, we analyzed the ROS production of neutrophils upon contact with *S. mutans* wild type, *deoC* mutant, and the complemented strain. As displayed in Figure 6A, the ROS production reached amaximum at approximately 2 h (MOI = 200) in all the tested strains, which was indicative that *S. mutans* was recognized by human neutrophils and had the potential to induce NET formation. ROS production during the observed time periods was shown by measuring the area under the curve. No significant difference was observed in the ROS levels between the wildtype, *deoC* mutant, and the complemented strain (Figure 6B), which indicated that the neutrophil activation potential of *S. mutans* was independent of DeoC.

NET formation was quantified using the fluorescent dye SytoxGreen, which detects extracellular DNA. In the case of neutrophils stimulated with *S. mutans* strains, the NET level increased over time with a lag-phase of 3 h, while only 1 h lag phase was observed for PMA treated neutrophils (Figure 6C). After 6 h of incubation, the fluorescence increased over time and reached a plateau at 47.54 and 62.24% of the total DNA when the neutrophils were stimulated with *deoC* mutant and PMA, respectively (Figures 6C,D). Notably, the fluorescence continued to increase after 6 h in the mutant stimulated neutrophils and kept steady after 9.5 h (Figure 6C). The sharp increase in DNA fluorescence from the 7th hour might be attributed to the death of the mutant cells and the subsequent release of DNA, since only 10% of the mutant cells survived after 4 h contact with NETs as revealed later in **Figure 8B**. However, wild-type-stimulated and complemented-strain-stimulated neutrophils exhibited only a slight increase in fluorescent signal. To confirm that the low fluorescence in the wild-type-stimulated neutrophils was due to nuclease activity, DNaseI was added to the PMA-treated neutrophils as a control. As expected, the fluorescent intensity decreased rapidly upon DNaseI addition (Figures 6D,E).

**Figure 7:**
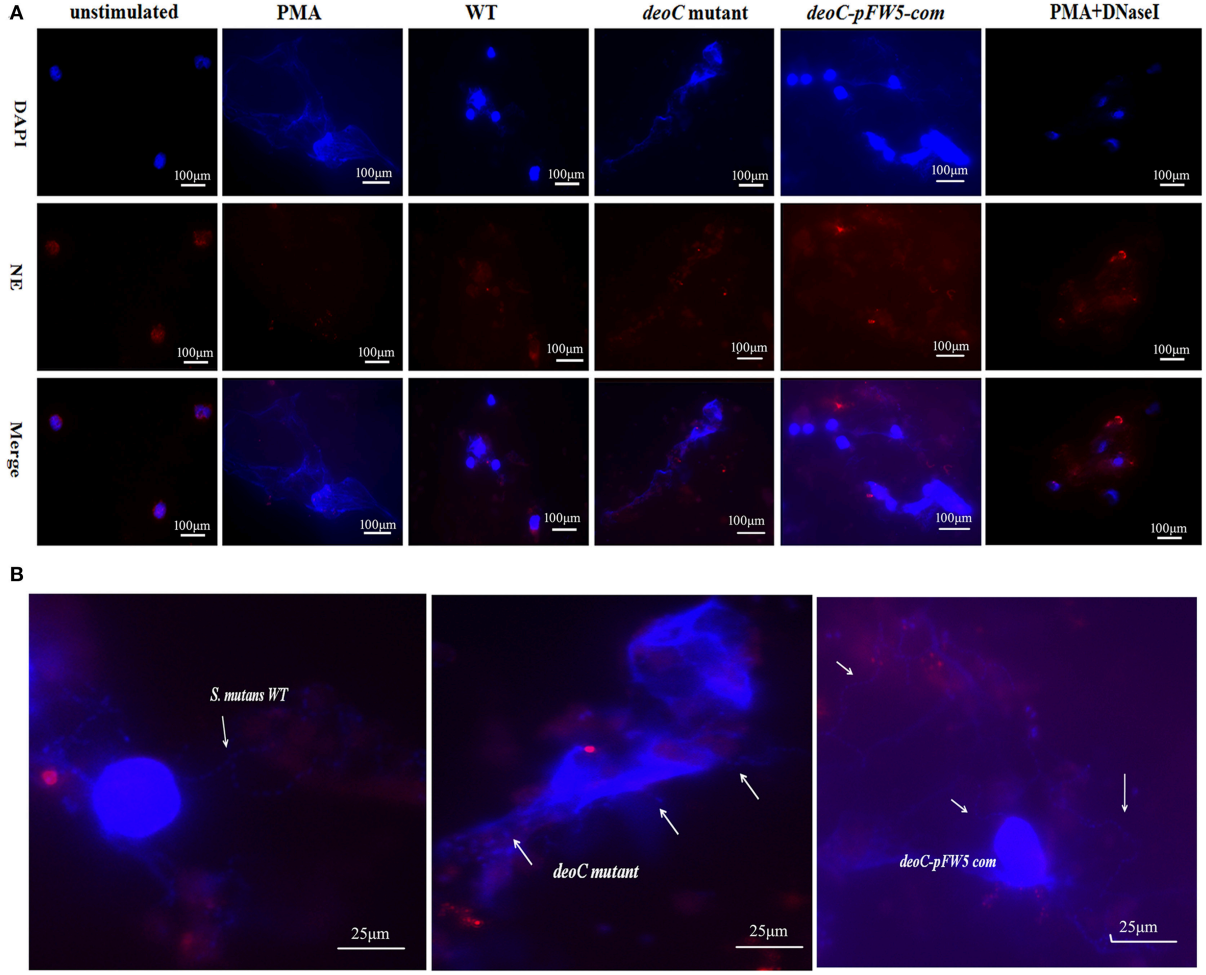
**Visualization of NET formation and degradation. (A)** Neutrophils were stimulated for 4 h either with PMA, *S. mutans* wild type (WT), *deoC* mutant, or pFW5-*deoC* com (MOI 200), or were left unstimulated. **(B)** Enlarged immunofluorescent micrographs of NE (HRP conjugated, red), DNA (DAPI, blue), and *S. mutans* WT, *deoC* mutant, and pFW5-*deoC*com strains; the arrows indicate the *S. mutans* cells.

To visualize NET formation induced by *S. mutans*, human neutrophils were stimulated with PMA, wild type, *deoC* mutant, and pFW5-*deoC*com. As a positive control, PMA-stimulated neutrophils exhibited web-like structures and the elastase was destroyed and scattered, compared with the intact neutrophils with nuclei and elastase within the cytoplasm in the unstimulated intact neutrophils (Figure 7A). After DNaseI treatment for 4 h, only deformed nuclei and scattered elastase could be detected, without DNA web-like structures. When co-cultured with the wild-type *S. mutans* or the complemented strain, no intact NETs were observed, characterized by the low intensity of DNA or the absence of DNA in areas of elastase. In addition, large areas of NE surrounded by *S. mutans* in an intact chain were visible (Figure 7B), suggestive of degradation of DNA in NETs by *S. mutans*. In comparison to the wild type, the *deoC*-mutant-stimulated neutrophils were associated with web-like DNA structures and dispersed elastase could be observed (Figure 7A), indicative of the incapability of the *deoC* mutant to degrade the DNA of the NETs, especially in comparison to the intact chain structure of wild type, the *deoC* mutant was found to be scattered in the DNA web (Figure 7B).

### *deoC* enhances survival of *S. mutans*in the presence of NETs

**Figure 8:**
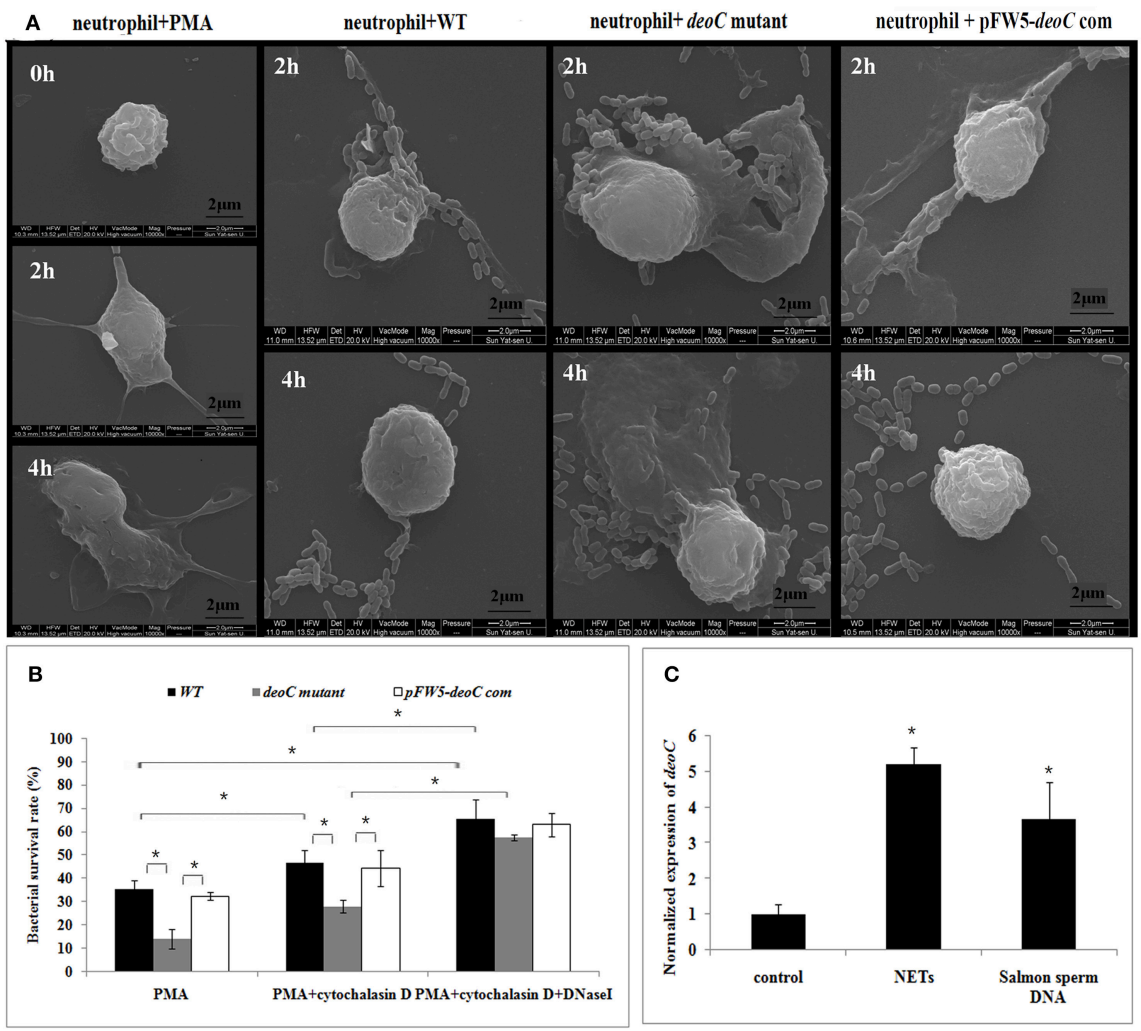
**DeoC facilitated survival of ***S. mutans*** from NETs.(A)** Scanning electron microscopy showed that web-like structures released by neutrophils were degraded by *S. mutans* wild type upon 4 h of co-culture, while the *deoC* mutant was trapped by NETs. **(B)** Survival rate of *S. mutans* subjected to killing by PMA-activated human neutrophils in the absence or presence of cytochalasin D and/or DNaseI. Bacterial survival is expressed as a percentage of the initial inoculum determined by CFU. **(C)** qRT-PCR analysis of *deoC* expression upon contact with neutrophils or extracellular DNA. Expression of the analyzed genes was compared with that of the control in the absence of NETs or extracellular DNA. ^*^P < 0.05.

Scanning electron microscope visualization of neutrophils revealed web-like structures trapping *S. mutans* wild-type cells after 2 h of co-culture, and the web-like structure disappeared after 4 h. In contrast, the *deoC* mutant got entrapped within NETs and no degradation of NETs was noted (Figure 8A). Due to antimicrobial effectors present at high concentrations in NETs, it was hypothesized that DeoC enhanced *S. mutans* survival in the presence of neutrophils. To test this hypothesis, a plate-based NET killing assay was performed. As shown in Figure 8B, a lower survival rate of *deoC* mutant was observed after incubation with PMA-stimulated neutrophils compared with the wild type and complemented strains (*P* < 0.05). In the presence of cytochalasin D or cytochalasinD+DNaseI, survival of all the *S. mutans* strains was increased (*P* < 0.05) in comparison with the PMA-treated neutrophils, suggestive of the role of phagocytic killing in neutrophils. However, a greater increase in survival of the *deoC* mutant was observed than of the wild type and the complemented strain, indicative that the DeoC activity of *S. mutans* was critical for the escape from NET-mediated extracellular killing.

To determine whether gene expression of nucleases was induced in the presence of NETs, qRT-PCR was employed to analyze the expression of *deoC* in *S. mutans*. It was found that *deoC* expression was up-regulated five-fold in *S. mutans* incubated with PMA-treated neutrophils. A similar induction was observed in *S. mutans* incubated with commercially available salmon sperm DNA (Figure 8C).

## Discussion

Growth in a biofilm plays an important role during infection by sheltering microbes from a range of stresses and clearance mechanisms, such as impeding the access of the immune system and antimicrobials (de laFuente-Nunez et al., 2013; Scherr et al., 2014). Beyond offering resistance to clearance mechanisms, biofilms also disperse cells to seed new sights of infection or mediate an acute infection (Lauderdale et al., 2010). The primary strategy employed by various bacterial species to actively disperse biofilm is the production of various exo-enzymes to degrade the extracellular polymericmatrix, including proteases and nucleases (Lister and Horswill, 2014). In this work, we identified a deoxyribonuclease encoded by *deoC* as the potential active factor regulating *S. mutans* biofilm dispersal. The role of *deoC* in biofilm dispersal was initially verified by the compromised ability of a *S. mutans deoC* mutant to disperse cells, although the ability was not completely lost. Since *S. mutans* possesses several other putative ribonucleases and endonucleases (Ajdic et al., 2002), we speculated that the partial biofilm dispersal ability of *deoC* mutant might be in part due to the existence of these nucleases. Similarly, *S. aureus* also produces two nucleases (Nuc and Nuc2), the activity of Nuc2 is very low and only able to partially disperse existing biofilms (Kiedrowski et al., 2014). In contrast, a recent study has shown that a ribonuclease of *Bacillus licheniformis* exhibits no dispersal efficacy in spite of the observation of it being within active supernatant (Nijland et al., 2010). Whether these ribonucleases and endonucleases in *S. mutans* had dispersal efficacy was still unknown due to the difficulty in studying them in wild-type backgrounds, as the low activity was masked by deoxyribonuclease (Kiedrowski et al., 2014).

During infection, dispersal of cells from the biofilm plays a key role in the communicable transmission of many pathogens to secondary sites and the worsening of the infection. In the oral cavity, *S. mutans* detached from dental biofilms in a mother's mouth can be transmitted to an infant (Berkowitz and Jones, 1985). Detached *S. mutans* cells can also be translocated to adjacent teeth through salivary flow (Svanberg and Loesche, 1978). Dispersed *S. mutans* from biofilm can easily gain access into the bloodstream to induce infective endocarditis through gingival wounds during dental surgery (Jung et al., 2015). Endogenous control of *S. mutans* spread is exerted by different host defense mechanisms, among them neutrophils are known to be a key host factor in the response to bacterial challenge. Activated neutrophils release NETs, which contain antimicrobial proteins bound to a DNA scaffold, for the clearance of the pathogens. To withstand these attacks, pathogens have evolved several mechanisms to circumvent neutrophil killing (Urban et al., 2006). Oral microbes often overcome host defenses and the reason why neutrophil or intravascular immunity fails to prevent *S. mutans* colonization was unclear. As a key feature of NET formation, the ROS production data presented herein demonstrated that *S. mutans* strains were recognized by human neutrophils and capable of stimulating NET formation independent of DeoC. The decreased NET integrity in response to *S. mutans* wild type and the complemented strain, therefore, can be attributed to the loss of DeoC, not to a reduced ability to stimulate NET formation. These findings established DeoC as a putative virulence factor in *S. mutans* defense against innate immunity. Secreted nucleases have been shown to digest the DNA scaffold of NETs, and aid in the escape from NETs to promote pathogen survival and spread in various species of *Streptococcus*. DNase Sda1 is both necessary and sufficient to promote groupA *Streptococcus* neutrophil resistance (Buchanan et al., 2006). *Streptococcus sanguinis* produces a wall-anchored nuclease, which contributes to its survival when encountering NETs (Morita et al., 2014). *Streptococcus suis* DNase SsnA contributes to degradation of NETs and evasion of NET-mediated antimicrobial activity (de Buhr et al., 2014). *Streptococcus pneumonia* degrades the NETs locally in the vicinity of trapped bacteria through a cell-surface-associated endonuclease encoded by *endA* (Beiter et al., 2006). These proteins share several characteristics, including a high degree of sequence similarity, being bacterial cell bounded endonucleases and typically possessing a signal peptide. DeoC-mediated NET degradation has two features that distinguish it from these proteins. First, DeoC cannot degrade DNA independently, but it is essential for DNA degradation activity. Considering that there are other putative ribonucleases and endonucleases, we suspect that DeoC may act somewhat synergistically with these putative nuclease released by *S. mutans*. Second, *deoC* lacks the signal sequence as predicted by the Protein Subcellular Localization Prediction Tool (data not shown), which excludes the possibility of active secretion of DeoC. It is, therefore, intriguing how DeoC gets outside of the cells and plays a role in NET degradation. Considering that the highest DNA degradation activity was observed during 48–60 h (Figure 3), the maturation stage of biofilm with substantial autolysis of biofilm cells (Perry et al., 2009), we assumed DeoC release may be due to the autolysis of *S. mutans* during infection.

Phagocytosis of microbes and formation of NETs are the two main antimicrobial strategies of neutrophils (Seper et al., 2013). NETs have been shown to mediate their antimicrobial activity through bacterial entrapment and subsequent direct bacterial killing by the antimicrobial peptides and histones that are embedded in the NETs.

*S. mutans*, like other oral streptococci, causes bacteremia most frequently in neutropenic patients (Marron et al., 2000) and is likely to be sensitive to phagocytic killing. The higher survival rate of *S. mutans* after phagocytosis inhibition suggested a role of phagocytosis in *S. mutant* killing. Upon DNaseI addition, the increase in the survival rate of the *deoC* mutant was indicative of a more important role of NET-mediated killing of *S. mutans*. What induced the production of nuclease during infection? It has been reported that one signal for induction of nucleases in mature biofilm might be nutrient limitation in biofilms (Seper et al., 2013). It could be possible that carbon limitation in mature biofilm induces the nuclease level of *S. mutans*; thus initiating the release of cells from biofilm. When entrapped by neutrophils, the presence of NETs or extracellular DNA might be an alternative signal for induction of the nucleases, as demonstrated in this study. Similar results have been obtained in *Vibrio cholerae*, of which nuclease encoding gene (*xds*) was characterized as the gene induced at late stages of the infection (Schild et al., 2007).

**Figure 9:**
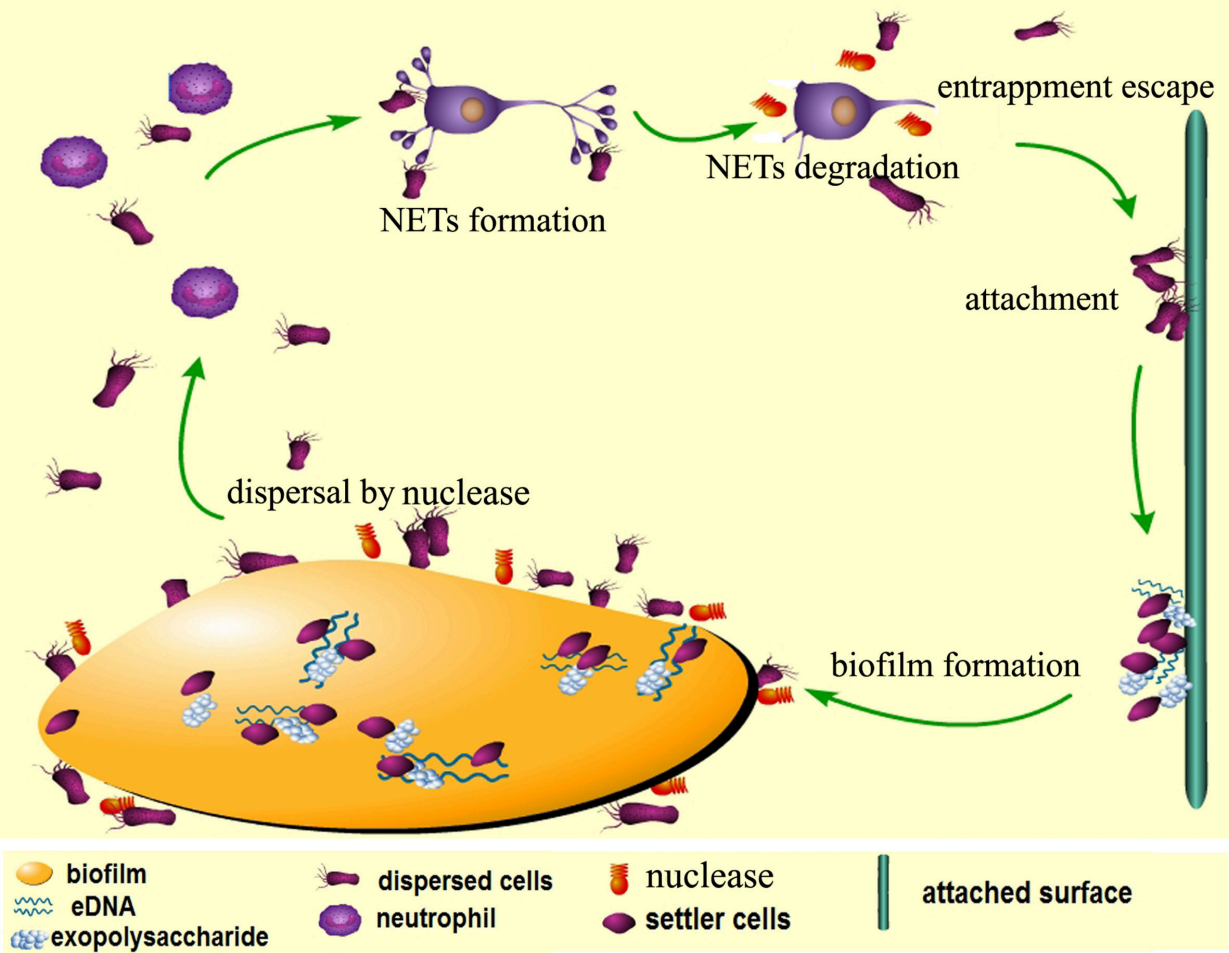
**Proposed model for nuclease-dependent ***S. mutans*** dispersal and escape from NETs**. When the biofilm is mature, expression of DeoC mediates degradation of eDNA (extracellular DNA) in the matrix to loosen the biofilm structure and the cells are released to the environment. The dispersed cells escape killing by the neutrophils through DeoC-mediated NET degradation and subsequently attach to new surfaces and form new biofilms.

The data obtained from this study assign two major roles for DeoC of *S. mutans* during infection, and we have proposed a model for nuclease-dependent *S. mutans* dispersal and escape from NETs (Figure 9). On one side, DeoC modulated biofilm dispersal, since a *deoC* mutant displays a defect in biofilm dispersal. On the other side, it plays a critical role in facilitating the escape of *S. mutans*, since DeoC mediates NET degradation and reduces NET-mediated killing.

## Author contributions

Experiments were performed by the following authors: Conceived and designed the experiments-JL and XW; Performed the experiments-JL, WL, LG and LS; Wrote the paper- JL; Revised the manuscript-JQL. The manuscript had been reviewed by all authors before submission.

## Funding

This work was supported by the National Natural Science Foundation of China under grant < 81500836, 81670982 >; Medical science and technology research fund of Guangdong Province China under grant < A2015190 >.

### Conflict of interest statement

The authors declare that the research was conducted in the absence of any commercial or financial relationships that could be construed as a potential conflict of interest.

## Abbreviations

CFU: colony forming unit
CSP: competence stimulating peptide
DEGs: differentially expressed genes
DR5P: 2-deoxyribose-5-phosphate
GO: gene ontology
LB: Luria–Bertani
MOI: multiplicity of infection
NE: neutrophil elastase
NET: neutrophil extracellular trap
PLS: polystyrene
PMA: phorbol 12-myristate 13-acetate
qRT-PCR: quantitative real-time PCR
ROS: reactive oxygen species
Spe: spectinomycin.
