# Supplementary material for: A Nuclease from Streptococcus mutans Facilitates Biofilm Dispersal and Escape from Killing by Neutrophil Extracellular Traps
Source: Front Cell Infect Microbiol. 2017 Mar 28;7:97. doi: 10.3389/fcimb.2017.00097 (PMC5368189; doi:10.3389/fcimb.2017.00097)

**Table S1.** Bacterial strains, plasmids, and primers used in the study

| **Bacterial strains** | **Major properties** | **Source or reference** |
| --- | --- | --- |
| *S. mutans*  WT | UA159 wild-type, serotype c, virulent strain of caries | Laboratory stock |
| *deoC* mutant  *E.coli* | UA159 derivative, deletion of *deoC*, Spe^r^ | This study |
| DH5α  BL21(DE3) | *endA1,hsdR17,supE44,recA1 (lacZYA-argF)*  *F^-^ ompT hsdS B(rB^-^mB^-^)dcm gal* (DE3) | Takara Biotechnology  Novagen |
| **Plasmids** |  |  |
| pFW5 | Cloning vector contains a spectinomycin-resistance marker (*aad9*) | Podbielski *et al*, 1996 |
| pFW5:LF^a^ | 800-bp DNA fragment with *deoC* flanked by upstream sequences cloned into pFW5, Spe^r^ | This study |
| pFW5-LF:RF^a^ | 800-bp DNA fragment wit h*deoC* flanked by downstream sequences cloned into pFW5:LF, Spe^r^ | This study |
| pET28a  pET*deoC* | Kan^r^ expression vector with 6His-tag coding sequence  pET derivative for expression 6His-DeoC | Novagen  This study |
| **Primers** | **DNA sequence(s) (5’-3’)** | **Purpose** |
| *deoC* LF^b^ | TCCCTAGCTGCACGATTTCC (Fw), | *deoC* left flank, |
|  | CGGTCGTGATGACAGTCGAA (Rev) |  |
| *deoC* RF^b^ | GCTCCCAGACGCATAGCAAA (Fw),  TTTGGGACAAGCTATCGGCA (Rev) | *deoC* right flank, |
| aad9  *deoC* | CTGATGTGAGAAGAGCCATTATGGA (Fw), | PCR  PCR |
|  | ATGGAGAAGATTCAGCCACTGC (Rev)  CCCAATACGTGTTGCACCTG ATTCGGGCAGTTGTTGAAGC |  |
| *dapA* | AAGGTTTGCTCTTAGCGGGA (Fw), | qRT-PCR |
|  | GGATGCTGAGCCAAACGAAG (Rev) |  |
| *SMU_1798c* | CCCCCGAAAAATCACGGTTG (Fw),  AAGCTGGTTTAGCAGGGCTT (Rev) | qRT-PCR |
| *infA* | AGCGGTATGTGATACGTCCA (Fw), | qRT-PCR |
|  | TGTCGAAACAATGCCAAATGC (Rev) |  |
| *scnK* | AAGCCCATACCGCTTCTTCT (Fw), | qRT-PCR |
|  | TTGGCACTGGCAGTCAACTC (Rev) |  |
| *clp* | CTGCGTTGCGGGATGTATTG (Fw)  AGCTCGGCGGACTAAAAACA (Rev) | qRT-PCR |
| *msmR* | AGGGGGTGTTGGCAAATTGA (Fw)  CCCTTGACTTAGCAGCAGGT (Rev) | qRT-PCR |
| *gtfD* | TGCAAGCGACGGAAAACAAG (Fw)  CTAGCGATACCCCAACGGTC(Rev) | qRT-PCR |
| *pknB* | GGGTCTTAAGGTGGGTGACG (Fw)  TCGAGGCTTCGCTTGATGTT (Rev) | qRT-PCR |
|  | TTCGCCAATCTGCTGGAACT (Fw)  ACTAACTATGGCGGCAGCTC (Rev) | qRT-PCR |
| SMU_500 | TCCCGTTACTCTTCGTGCAG (Fw)  TAAAGACATGTGCCGCTGGT (Rev) | qRT-PCR |
| SMU_984 | TTTGGCTTTGATGGCAATGGT (Fw)  GCTGGGCATTTCACTCACAA (Rev) | qRT-PCR |
| SMU_444 | GTCAGTTGGCATGGGAGTGA (Fw)  AGGTGAAAGGTTTGTTCTTTAGAC (Rev) | qRT-PCR |
| *glnQ* | GGGTATGCTATCTGGCGGAC (Fw)  GTGAGTGACGACGACCATGT (Rev) | qRT-PCR |
| *SMU_1316c* | CCCTGCTTTTACCAGCTGTC (Fw)  TCCTGCCCAGCCAAATAACA (Rev) | qRT-PCR |
| 16S rRNA | GCGACGATACATAGCCGACCT (Fw), | qRT-PCR |
|  | TCCATTGCCGAAGATTCCCTA (Rev) |  |

^a^ LF, RF: left flank, right flank; consisted of upstream and downstream sequences of *deoC*, respectively. All primers were designed with primer-BLAST and obtained from Invitrogen Biotechnology.

**Table S2. List of up-regulated genes in 96h *VS* 60h biofilm**

| **Locus tag** | **Gene name** | **NCBI annotation** | **Fold change** |
| --- | --- | --- | --- |
| SMU.1709 | *trkH* | putative potassium uptake protein TrkH | 1.52 |
| SMU.629 | *sod* | putative manganese-type superoxide dismutase, Fe/Mn-SOD | 1.68 |
| SMU.1142c | *spxA* | transcriptional regulator Spx | 1.60 |
| SMU.332 |  | hypothetical protein | 1.56 |
| SMU.379 |  | hypothetical protein | 1.54 |
| SMU.388 |  | putative integral membrane protein; branched-chain amino acid permease | 1.50 |
| SMU.2052c |  | hypothetical protein | 1.75 |
| SMUr04 |  | 16S ribosomal RNA | 3.55 |
| SMU.714 | *tuf* | elongation factor Tu | 1.53 |
| SMU.558 | *ileS* | isoleucyl-tRNA synthetase | 1.54 |
| SMU.1386 | *urk* | uridine kinase | 1.55 |
| SMU.1207 | *fic* | mobilization/cell filamentation proteins | 1.50 |
| SMUr01 |  | 16S ribosomal RNA | 3.73 |
| SMU.484 | *pknB* | putative serine/threonine protein kinase | 1.62 |
| SMU.1118c |  | putative ABC sugar transporter, permease protein | 1.72 |
| SMU.1563 | *pacL* | putative cation-transporting P-type ATPase PacL | 1.80 |
| SMU.233 | *ilvC* | ketol-acid reductoisomerase | 1.72 |
| SMU.589 |  | putative DNA-binding protein | 1.51 |
| SMU.1066 | *guaA* | GMP synthase | 1.56 |
| SMU.910 | *gtfD* | glucosyltransferase-S | 1.61 |
| SMU.990 | *dapA* | dihydrodipicolinate synthase | 1.69 |
| SMU.864 |  | putative ABC transporter, permease component | 1.64 |
| SMU.2028 | *sacB* | levansucrase precursor; beta-D-fructosyltransferase | 1.77 |
| SMU.1141c |  | hypothetical protein | 1.57 |
| SMU.588 |  | hypothetical protein | 1.56 |
| SMU.381c |  | hypothetical protein | 1.61 |

**Table S3. List of down-regulated genes in 96h *VS* 60h biofilm**

| **Locus tag** | **Gene name** | **NCBI annotation** | **Fold change** |
| --- | --- | --- | --- |
| SMU.440 |  | hypothetical protein | 1.93 |
| SMUt15 |  | tRNA-Phe | 14.39 |
| SMUt01 |  | tRNA-Ala | 1.66 |
| SMUt31 |  | tRNA-His | 3.83 |
| SMU.1146c |  | putative response regulator; homolog of RumR and ScnR | 1.58 |
| SMU.1287 |  | transcriptional regulator | 1.77 |
| SMU.500 |  | putative ribosome-associated protein | 2.36 |
| SMU.1814 | *scnK* | putative histidine kinase, ScnK-like protein | 1.82 |
| SMU.796 |  | hypothetical protein | 2.61 |
| SMUr03 |  | 5S ribosomal RNA | 2.48 |
| SMUt20 |  | tRNA-Thr | 7.47 |
| SMU.984 |  | hypothetical protein | 6.42 |
| SMU.1361c |  | TetR family transcriptional regulator | 4.94 |
| SMU.1157c |  | hypothetical protein | 1.92 |
| SMUt13 |  | tRNA-Ser | 3.42 |
| SMUt03 |  | tRNA-Asp | 12.46 |
| SMU.1024c |  | putative putative transposase | 1.62 |
| SMUt61 |  | tRNA-Cys | 19.04 |
| SMUt18 |  | tRNA-Ser | 6.05 |
| SMUt32 |  | tRNA-Gln | 2.83 |
| SMU.991 |  | putative ribonucleotide reductase | 1.52 |
| SMU.2160 |  | transmembrane protein | 2.13 |
| SMU.1977c |  | putative transcriptional regulator | 2.12 |
| SMU.528c |  | hypothetical protein | 1.74 |
| SMU.1106c |  | phosphoglycerate mutase-like protein | 1.96 |
| SMU.800 |  | hypothetical protein | 2.75 |
| SMU.1752c |  | hypothetical protein | 1.57 |
| SMUt14 |  | tRNA-Met | 3.15 |
| SMU.593 | *furR* | putative ferric uptake regulator protein FurR | 2.49 |
| MU.510c |  | hypothetical protein | 1.55 |
| SMU.1519 | *glnQ* | putative amino acid ABC transporter, ATP-binding protein | 3.73 |
| SMUt28 |  | tRNA-Phe | 14.35 |
| SMU.31 |  | hypothetical protein | 2.77 |
| SMU.956 | *clp* | putative Clp-like ATP-dependent protease, ATP-binding subunit | 2.37 |
| SMUt30 |  | tRNA-Trp | 7.80 |
| SMUt33 |  | tRNA-Leu | 5.33 |
| SMUt19 |  | tRNA-Leu | 8.93 |
| SMU.1776c |  | hypothetical protein | 3.58 |
| SMU.1070c |  | hypothetical protein | 4.94 |
| SMUr02 |  | 23S ribosomal RNA | 2.29 |
| SMU.444 |  | hypothetical protein | 2.10 |
| SMUt52 |  | tRNA-OTHER | 4.30 |
| SMU.1062 | *opuAb* | putative ABC transporter, proline/glycine betaine permease protein | 1.71 |
| SMUt25 |  | tRNA-Glu | 12.50 |
| SMU.1847 | *efp* | elongation factor P | 2.15 |
| SMU.735 |  | hypothetical protein | 2.60 |
| SMUt17 |  | tRNA-Ile | 3.39 |
| SMU.1914c |  | hypothetical protein | 2.04 |
| SMU.1872c |  | hypothetical protein | 3.97 |
| SMU.342 |  | hypothetical protein | 1.60 |
| SMU.88c |  | mechanosensitive ion channel | 2.28 |
| SMU.682 |  | hypothetical protein | 2.28 |
| SMU.1955 | *groES* | co-chaperonin GroES | 2.78 |
| SMUt08 |  | tRNA-Leu | 2.36 |
| SMU.1030 |  | putative polyribonucleotide nucleotidyltransferase; Tn916 ORF8-like | 1.95 |
| SMUt04 |  | tRNA-Lys | 1.75 |
| SMU.172 |  | putative cell growth regulatory protein | 1.63 |
| SMU.1774c |  | hypothetical protein | 2.22 |
| SMU.1360c |  | hypothetical protein | 1.90 |
| SMU.1246c |  | putative transcriptional regulator | 2.30 |
| SMU.1027 |  | putative transcription regulator | 6.21 |
| SMUt10 |  | tRNA-Pro | 1.70 |
| SMU.2155 |  | hypothetical protein | 3.82 |
| SMU.1650 | *end3* | putative endonuclease III (DNA repair) | 1.70 |
| SMU.1956c |  | hypothetical protein | 1.69 |
| SMU.296 |  | hypothetical protein | 2.01 |
| SMUt07 |  | tRNA-Gly | 4.32 |
| SMU.879 | *msmF* | multiple sugar-binding ABC transporter, permease protein MsmF | 1.66 |
| SMU.1291c |  | hypothetical protein | 3.83 |
| SMU.186 | *sloR* | putative metal-dependent transcriptional regulator | 2.22 |
| SMU.2104a | *rpmF* | 50S ribosomal protein L32 | 1.69 |
| SMUt40 |  | tRNA-Arg | 2.41 |
| SMU.925 |  | hypothetical protein | 1.85 |
| SMU.166 |  | hypothetical protein | 3.35 |
| SMUt11 |  | tRNA-Met | 5.51 |
| SMU.1072c |  | putative acetyltransferase | 1.77 |
| SMU.231 | *ilvB* | acetolactate synthase catalytic subunit | 1.72 |
| SMU.405c |  | putative transcriptional regulator | 2.53 |
| SMU.1783 | *proS* | prolyl-tRNA synthetase | 2.47 |
| SMU.1573 | *metK* | S-adenosylmethionine synthetase | 1.55 |
| SMUr05 |  | 23S ribosomal RNA | 2.25 |
| SMUt35 |  | tRNA-Leu | 6.22 |
| SMU.609 |  | putative 40K cell wall protein precursor | 1.86 |
| SMUt34 |  | tRNA-Ser | 4.44 |
| SMU.814 | *mutT* | putative MutT-like protein | 1.76 |
| SMU.1794c |  | hypothetical protein | 1.73 |
| SMU.1927 |  | putative ABC transporter, ATP-binding protein | 1.57 |
| SMU.840c |  | hypothetical protein | 2.78 |
| SMU.590c |  | putative transposase, fragment | 1.72 |
| SMU.1399 |  | hypothetical protein | 2.41 |
| SMU.164 |  | putative tRNA/rRNA methyltransferase | 1.83 |
| SMU.2151 | *pgsA* | putative phosphotidylglycerophosphate synthase | 2.33 |
| SMUt37 |  | tRNA-Arg | 3.24 |
| SMU.560c |  | hypothetical protein | 2.01 |
| SMU.1618 | *dagK* | diacylglycerol kinase | 1.67 |
| SMU.1957 |  | putative PTS system, mannose-specific IID component | 4.55 |
| SMU.125 |  | hypothetical protein | 1.91 |
| SMUt09 |  | tRNA-Arg | 2.03 |
| SMU.1628 |  | hypothetical protein | 1.77 |
| SMUt16 |  | tRNA-Gly | 4.76 |
| SMU.1870 | *mutS2* | putative DNA mismatch repair protein MutS2 | 1.64 |
| SMU.167 |  | hypothetical protein | 4.53 |
| SMU.506 |  | putative type II restriction endonuclease | 1.67 |
| SMU.1512 | *pheS* | phenylalanyl-tRNA synthetase subunit alpha | 1.63 |
| SMU.1647c |  | putative transcriptional regulator | 2.00 |
| SMU.136c |  | putative transcriptional regulator | 2.01 |
| SMUt38 |  | tRNA-Gln | 2.83 |
| SMUt29 |  | tRNA-Tyr | 1.54 |
| SMU.2098 | *argS* | arginyl-tRNA synthetase | 2.05 |
| SMU.669c |  | putative glutaredoxin | 1.70 |
| SMU.494 |  | fructose-6-phosphate aldolase | 1.86 |
| SMU.236c |  | putative transcriptional regulator | 2.05 |
| SMUt51 |  | tRNA-Thr | 24.85 |
| SMU.1123 | *deoC* | deoxyribose-phosphate aldolase | 4.84 |
| SMU.1721c |  | putative diaminopimelate decarboxylase | 1.65 |
| SMU.2060 |  | LysR family transcriptional regulator | 1.76 |
| SMU.1378 |  | hypothetical protein | 2.00 |
| SMU.1116c |  | hypothetical protein | 2.15 |
| SMUt06 |  | tRNA-Thr | 2.62 |
| SMUt43 |  | tRNA-Leu | 2.16 |
| SMUt05 |  | tRNA-Leu | 2.46 |
| SMU.1803c |  | hypothetical protein | 3.07 |
| SMU.709 |  | hypothetical protein | 1.71 |
| SMU.1780 | *recX* | recombination regulator RecX | 1.67 |
| SMU.876 | *msmR* | putative MSM operon regulatory protein | 2.18 |
| SMU.1855 |  | hypothetical protein | 2.06 |
| SMU.1125c |  | hypothetical protein | 1.60 |
| SMU.336 | *rnpA* | ribonuclease P | 1.58 |
| SMU.2096c |  | hypothetical protein | 3.47 |
| SMU.1455 | *mutX* | mutator protein, pyrophosphohydrolase | 1.73 |
| SMU.508 |  | hypothetical protein | 1.84 |
| SMU.2061 |  | hypothetical protein | 2.78 |
| SMU.2048 |  | hypothetical protein | 1.70 |
| SMUr13 |  | 5S ribosomal RNA | 2.56 |

**Table S4. List of up-regulated genes in 60h *VS* 48h biofilm**

| **Locus tag** | **Gene name** | **NCBI annotation** | **Fold change** |
| --- | --- | --- | --- |
| SMU.1602 |  | putative NAD(P)H-flavin oxidoreductase | 1.55 |
| SMU.1709 | *trkH* | putative potassium uptake protein TrkH | 1.69 |
| SMU.333 |  | hypothetical protein | 1.61 |
| SMU.422 | *rbfA* | ribosome-binding factor A | 1.53 |
| SMU.1665 | *livF* | putative branched chain amino acid ABC transporter, ATP-binding protein | 1.72 |
| SMU.1346 | *bacT* | putative thioesterase BacT | 1.92 |
| SMU.1895c |  | hypothetical protein | 1.59 |
| SMU.379 |  | hypothetical protein | 1.81 |
| SMU.2052c |  | hypothetical protein | 1.90 |
| SMU.277 |  | hypothetical protein | 1.55 |
| SMUr04 |  | 16S ribosomal RNA | 3.67 |
| SMU.1434c |  | putative glycosyltransferase | 1.52 |
| SMU.2004 | *infA* | translation initiation factor IF-1 | 1.55 |
| SMU.1207 | *fic* | mobilization/cell filamentation proteins | 1.54 |
| SMU.1922 | *dnaB* | putative chromosome replication protein | 1.75 |
| SMU.1288 | *rplS* | 50S ribosomal protein L19 | 1.60 |
| SMU.2149c | *cbiO* | cobalt transporter ATP-binding subunit | 1.53 |
| SMUr01 |  | 16S ribosomal RNA | 3.79 |
| SMU.1384 | *leuA* | 2-isopropylmalate synthase | 1.60 |
| SMU.1798c |  | hypothetical protein | 1.55 |
| SMU.990 | *dapA* | dihydrodipicolinate synthase | 1.67 |
| SMU.1771c |  | hypothetical protein | 1.96 |
| SMU.2028 | *sacB* | levansucrase precursor; beta-D-fructosyltransferase | 1.58 |
| SMU.389 |  | hypothetical protein | 1.53 |
| SMU.1902c |  | hypothetical protein | 1.70 |
| SMU.1730c |  | putative acetyltransferase | 1.67 |
| SMU.248 |  | putative ABC transporter, membrane protein | 1.80 |
| SMU.1432c |  | putative endoglucanase precursor | 1.56 |
| SMU.588 |  | hypothetical protein | 1.62 |
| SMU.1846c |  | hypothetical protein | 1.54 |

**Table S5. List of down-regulated genes in 60h *VS* 48h biofilm**

| **Locus tag** | **Gene name** | **NCBI annotation** | **Fold change** |
| --- | --- | --- | --- |
| SMU.1168 |  | putative transcriptional regulator | 1.54 |
| SMU.2097 |  | hypothetical protein | 1.65 |
| SMU.440 |  | hypothetical protein | 1.50 |
| SMUt31 |  | tRNA-His | 1.51 |
| SMU.1287 |  | putative transcriptional regulator | 2.05 |
| SMU.1041 |  | putative ABC transporter, ATP-binding protein | 1.61 |
| SMU.500 |  | putative ribosome-associated protein | 2.16 |
| SMU.1814 | *scnK* | putative histidine kinase, ScnK-like protein | 1.83 |
| SMU.796 |  | hypothetical protein | 1.59 |
| SMUr03 |  | 5S ribosomal RNA | 1.91 |
| SMU.984 |  | hypothetical protein | 2.82 |
| SMU.1361c |  | TetR family transcriptional regulator | 2.14 |
| SMU.1157c |  | hypothetical protein | 1.62 |
| SMUt13 |  | tRNA-Ser | 1.57 |
| SMU.1024c |  | putative putative transposase | 1.72 |
| SMUt61 |  | tRNA-Cys | 4.74 |
| SMUt18 |  | tRNA-Ser | 2.66 |
| SMUt32 |  | tRNA-Gln | 1.78 |
| SMU.2156 | *recF* | recombination protein F | 1.51 |
| SMU.465 | *nadE* | NAD synthetase | 1.69 |
| SMU.845 |  | hypothetical protein | 1.63 |
| SMU.593 | *furR* | putative ferric uptake regulator protein FurR | 2.15 |
| SMU.173 |  | putative ppGpp-regulated growth inhibitor | 1.71 |
| SMU.510c |  | hypothetical protein | 1.55 |
| SMU.866 |  | hypothetical protein | 1.55 |
| SMU.1519 | *glnQ* | putative amino acid ABC transporter, ATP-binding protein | 1.60 |
| SMU.956 | *clp* | putative Clp-like ATP-dependent protease, ATP-binding subunit | 2.22 |
| SMUt30 |  | tRNA-Trp | 4.47 |
| SMU.168 |  | putative transcriptional regulator | 1.63 |
| SMUt33 |  | tRNA-Leu | 1.69 |
| SMUt36 |  | tRNA-Ser | 1.93 |
| SMUt19 |  | tRNA-Leu | 2.84 |
| SMU.1070c |  | hypothetical protein | 2.13 |
| SMUr02 |  | 23S ribosomal RNA | 1.67 |
| SMU.444 |  | hypothetical protein | 2.81 |
| SMU.2107c |  | hypothetical protein | 1.56 |
| SMUt52 |  | tRNA-OTHER | 1.63 |
| SMU.1088 | *apbE* | putative thiamine biosynthesis lipoprotein | 1.55 |
| SMU.1062 | *opuAb* | putative ABC transporter, proline/glycine betaine permease protein | 1.54 |
| SMUt25 |  | tRNA-Glu | 2.24 |
| SMU.1847 | *efp* | elongation factor P | 1.57 |
| SMU.735 |  | hypothetical protein | 1.83 |
| SMUt17 |  | tRNA-Ile | 2.50 |
| SMU.1872c |  | hypothetical protein | 2.40 |
| SMU.88c |  | mechanosensitive ion channel | 2.13 |
| SMU.682 |  | hypothetical protein | 1.73 |
| SMU.1030 |  | putative polyribonucleotide nucleotidyltransferase; Tn916 ORF8-like | 2.03 |
| SMU.172 |  | putative cell growth regulatory protein | 1.80 |
| SMU.1774c |  | hypothetical protein | 1.66 |
| SMU.427 | *copZ* | putative copper chaperone | 1.67 |
| SMU.1246c |  | putative transcriptional regulator | 2.05 |
| SMU.1027 |  | putative transcription regulator | 2.05 |
| SMU.2155 |  | hypothetical protein | 1.63 |
| SMU.1650 | *end3* | putative endonuclease III (DNA repair) | 1.56 |
| SMU.1956c |  | hypothetical protein | 1.77 |
| SMU.1687 | *ppaC* | putative manganese-dependent inorganic pyrophosphatase | 1.71 |
| SMU.296 |  | hypothetical protein | 1.59 |
| SMUt07 |  | tRNA-Gly | 1.58 |
| SMU.879 | *msmF* | multiple sugar-binding ABC transporter, permease protein MsmF | 2.34 |
| SMU.1291c |  | hypothetical protein | 2.59 |
| SMU.186 | *sloR* | putative metal-dependent transcriptional regulator | 2.18 |
| SMUt40 |  | tRNA-Arg | 2.24 |
| SMU.166 |  | hypothetical protein | 2.48 |
| SMUt11 |  | tRNA-Met | 2.24 |
| SMU.1072c |  | putative acetyltransferase | 1.74 |
| SMU.231 | *ilvB* | acetolactate synthase catalytic subunit | 1.76 |
| SMU.405c |  | putative transcriptional regulator | 2.12 |
| SMU.1783 | *proS* | prolyl-tRNA synthetase | 1.75 |
| SMU.896 |  | hypothetical protein | 2.45 |
| SMUr05 |  | 23S ribosomal RNA | 1.67 |
| SMUt12 |  | tRNA-Met | 1.89 |
| SMU.609 |  | putative 40K cell wall protein precursor | 2.31 |
| SMUt34 |  | tRNA-Ser | 2.80 |
| SMU.814 | *mutT* | putative MutT-like protein | 1.55 |
| SMU.1794c |  | hypothetical protein | 1.65 |
| SMU.840c |  | hypothetical protein | 1.67 |
| SMU.503c |  | hypothetical protein | 1.95 |
| SMU.590c |  | putative transposase, fragment | 1.68 |
| SMU.745 | *lmrB* | putative drug-export protein; multidrug resistance protein | 1.51 |
| SMU.560c |  | hypothetical protein | 1.69 |
| SMU.1957 |  | putative PTS system, mannose-specific IID component | 2.85 |
| SMUt16 |  | tRNA-Gly | 1.90 |
| SMU.1870 | *mutS2* | putative DNA mismatch repair protein MutS2 | 1.58 |
| SMU.167 |  | hypothetical protein | 2.92 |
| SMU.506 |  | putative type II restriction endonuclease | 1.53 |
| SMU.934 |  | putative amino acid ABC transporter, permease protein | 1.52 |
| SMU.1073 | *fthS* | formate--tetrahydrofolate ligase | 1.61 |
| SMUt38 |  | tRNA-Gln | 1.78 |
| SMU.632 |  | putative transcriptional regulator | 1.56 |
| SMU.2098 | *argS* | arginyl-tRNA synthetase | 1.84 |
| SMU.669c |  | putative glutaredoxin | 1.53 |
| SMUt51 |  | tRNA-Thr | 1.58 |
| SMU.1123 | *deoC* | deoxyribose-phosphate aldolase | 2.11 |
| SMU.1721c |  | putative diaminopimelate decarboxylase | 2.08 |
| SMU.1768c |  | hypothetical protein | 1.54 |
| SMU.1378 |  | hypothetical protein | 1.89 |
| SMUt05 |  | tRNA-Leu | 1.63 |
| SMU.1803c |  | hypothetical protein | 1.93 |
| SMU.897 |  | putative type I restriction-modification system, helicase subunits | 2.15 |
| SMUt62 |  | tRNA-Lys | 1.54 |
| SMU.876 | *msmR* | putative MSM operon regulatory protein | 2.08 |
| SMU.1855 |  | hypothetical protein | 1.97 |
| SMU.2096c |  | hypothetical protein | 1.62 |
| SMU.1455 | *mutX* | mutator protein, pyrophosphohydrolase | 1.70 |
| SMU.508 |  | hypothetical protein | 1.63 |
| SMU.2061 |  | hypothetical protein | 2.16 |
| SMUr13 |  | 5S ribosomal RNA | 1.96 |

Figure S1. PCR analysis of *deoC* mutant and p FW5-*deoC*com with primers of *deoC* ORF (1,2 and 3) and the flank regions (4, 5 and 6). 1and 4: S. mutans WT; 2 and 5: p FW5-*deoC*com; 3 and 6: *deoC* mutant


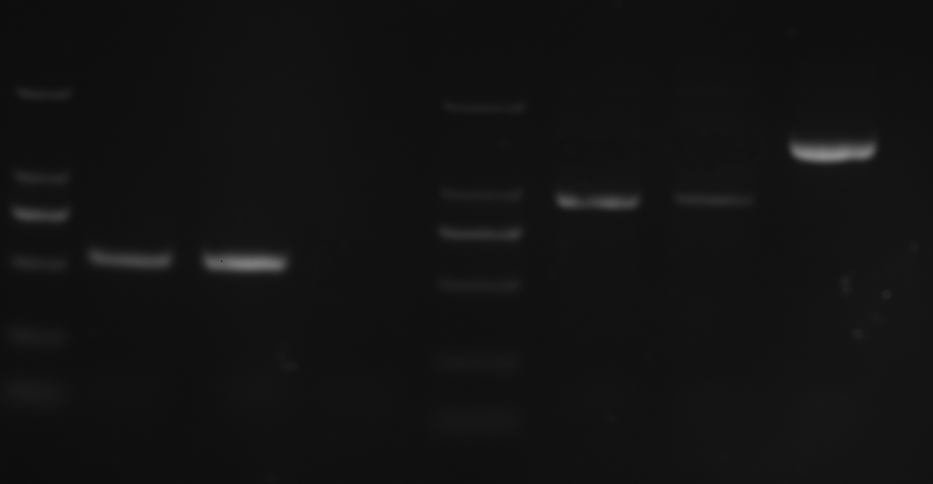


**M 1 2 3**

**M 4 5 6**

**2000**

**1000**

**750**

**500**

**250**

**100**

Figure S2 Quantitative RT-PCR verification of the relative expression levels of randomly selected DEGs and 16S rRNA was used for normalization. White: 60h *VS* 48h; Grey: 96h *VS* 60h. The value on the bars indicated the expression level determined by microarray. Most of the selected genes showed a similar expression trend, supporting a strong level of confidence in RNA-Seq data.


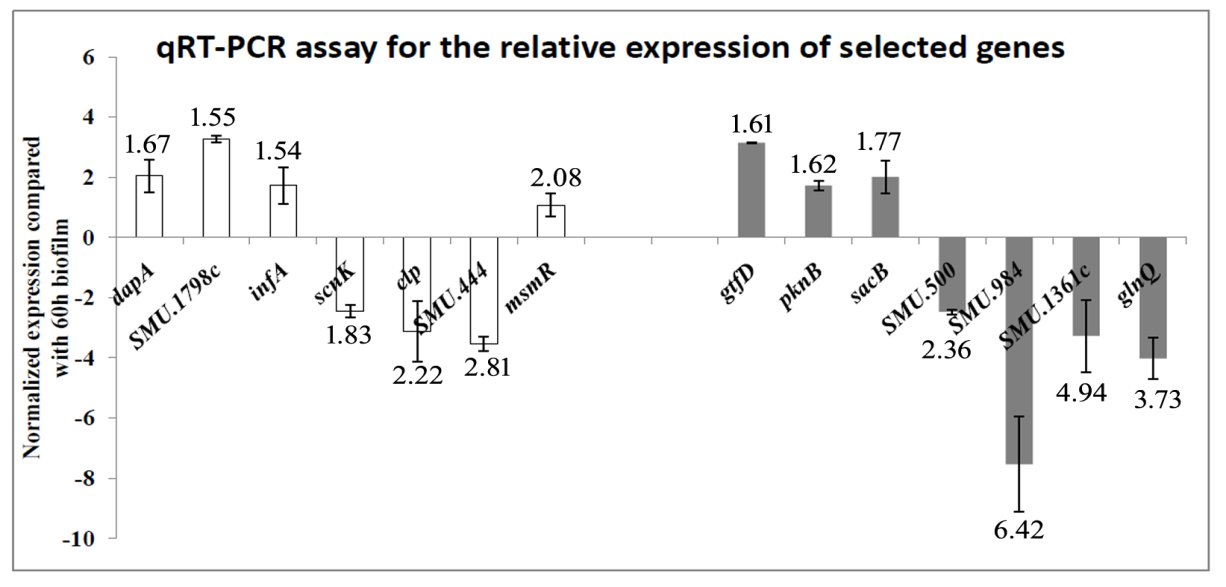

Supplement: Supplementary file 1 [file DataSheet1.docx]
